# Parental experiences of eczema advice in online parenting forums: a qualitative interview study

**DOI:** 10.3399/BJGPO.2022.0155

**Published:** 2023-05-17

**Authors:** Bethan Mair Treadgold, Ingrid Muller, Emma Teasdale, Neil Coulson, Amanda Roberts, Miriam Santer

**Affiliations:** 1 Primary Care Group, College of Medicine and Health, University of Exeter, Exeter, UK; 2 Primary Care Research Centre, University of Southampton, Southampton, UK; 3 Nottingham Centre for Public Health and Epidemiology, School of Medicine, Faculty of Medicine and Health Sciences, University of Nottingham, Nottingham, UK; 4 Centre of Evidence-Based Dermatology, Faculty of Medicine and Health Sciences, University of Nottingham, Nottingham, UK

**Keywords:** dermatology, eczema, child health, qualitative research, primary healthcare, general practice

## Abstract

**Background:**

Parents of children with eczema are increasingly turning to online parenting forums for advice about how to manage it. Little is known about how parents make sense of advice about eczema treatments in online forums, and how it affects their management of childhood eczema.

**Aim:**

To explore how parents of children with eczema make sense of and act on advice about eczema treatments exchanged in online parenting forums.

**Design & setting:**

Qualitative interviews with parents of children with eczema from the UK.

**Method:**

Fifteen parents were recruited through online advertisements and snowball sampling. Semi-structured interviews were carried out face to face or by telephone, and analysed inductively using reflexive thematic analysis.

**Results:**

When seeking advice from online parenting forums about eczema treatments, parents described appraising the credibility of advice and considering the potential suitability of treatments that were recommended in the forum. Parents proceeded to make sense of online advice through either reading advice and not engaging in online discussions, or actively engaging in online discussions to direct topics and seek most relevant advice. Parents discussed advice received online in subsequent consultations with their GP and requested prescriptions of recommended treatments. Some parents described trying new treatments without consulting their GP.

**Conclusion:**

Understanding how parents appraise, make sense of, and act on online advice could support healthcare professionals to disseminate advice appropriately, ask additional questions, and signpost parents to reliable online resources.

## How this fits in

Before this research, little was known about how parents and carers of children with eczema make sense of and act on advice about eczema treatments exchanged in online parenting forums. Findings from this research could support primary care health professionals in consultations to disseminate advice appropriately, ask additional questions, and signpost parents and carers to reliable online resources.

## Introduction

There has been a rapid increase in the use of online forums (otherwise known as online support communities, online support groups, and social media websites) for health-related purposes in the past two decades.^1^ Parents and other primary caregivers (hereafter referred to as ‘parents’) have been shown to place high value on health information rooted in the lived experiences of those living with or caring for others with health conditions, and that accounts of these experiences are readily accessible using online parenting forums.^2^ The wealth of social support exchanged in online parenting forums has been found to reduce parents’ fears, isolation, and worries when caring for an unwell child, and to increase parents’ confidence in caring for their child.^2,3^

Eczema (also known as atopic dermatitis) is a common condition in childhood, affecting 11–20% of children in the UK,^3^ most commonly in children aged ≤2 years.^4^ Eczema management involves applying topical treatments, such as emollient moisturisers and topical corticosteroids, and avoiding irritants and allergens.^5^ Management of eczema is challenging and time-consuming. The consequences of living with the condition, such as continuous itching, rashes, sleep disturbances, and the regimen of applying treatment, has been consistently found to have a considerable effect on the quality of life for children and their families.^6,7^ Parents’ beliefs around treatments for eczema have been found to be a barrier to treatment adherence,^8^ such as the inconvenience of treatment regimens, concerns about potential side effects, and not perceiving treatment recommendations to be important. Digital applications containing eczema advice have been found to vary in their medical accuracy and safety.^9^ Little research has been conducted exploring the experiences of parents of children with eczema in online parenting forums around advice about eczema management. Some parents of children with eczema have reported positive experiences, which include successfully obtaining desired advice, deciding whether online advice is useful to them or not, and benefiting emotionally from the supportive online presence of other parents of children with eczema.^10^ However, other parents have described the online world as bewildering owing to the volume of available eczema advice.^10^ In online parenting forums, parents have expressed diverse beliefs and uncertainties about using and applying topical corticosteroids to treat eczema,^11^ and about allergy testing for eczema.^12^ Yet, how parents of children with eczema make sense of and potentially use and act on treatment advice received from online parenting forums, and the consequences of this on the management of their child’s eczema, has not been explored.

Research among parents of children with other conditions has shown that many do not feel confident in their ability to appraise the trustworthiness of online advice, yet little is known about the specific appraisal processes of parents, nor how they made sense of, understood, and applied information from the online world.^13,14^ People managing their own health and illness appear to consider various aspects of the advice and website before adopting online advice, including the following: the owners and/or sponsors of websites; whether there was consensus across multiple information resources; characteristics of writing and language; adverts on websites; content authorship; and the design of websites.^15^ The wider literature has also reviewed how people have acted on advice obtained from the online world, including through making decisions about their treatments,^16^ making appointments with health professionals,^16,17^ and learning terminology to form questions and to know which treatments to request in medical consultations.^14,16^ However, given the array of challenges experienced by parents caring for children with eczema, it is possible that their processes of making sense of and acting on advice received from the online world may be different. This is unknown.

The aim of this study was to explore how parents of children with eczema make sense of and act on advice about eczema treatments exchanged in online parenting forums.

## Method

### Design

Semi-structured individual interviews were carried out face to face or by telephone.

### Sample and recruitment strategy

The study sought to recruit participants aged ≥18 years, residing in the UK, who were the parent or primary carer of a child with eczema. Participants were recruited through advertising the study in popular online parenting forums and social media websites, and opportunistically by inviting parents who had taken part in other eczema research by the research team, and through snowball sampling.

### Interview materials and procedure

An interview guide was developed by the research team, informed by the aims of the study and existing research. Questions explored which treatments participants sought advice about online, the process of deciding what advice to look at online, treatment advice exchanged in online parenting forums, and using the advice (see Box 1 for interview guide details). Interviews were carried out by author BT, a research psychologist with experience of qualitative research, with parents of children with eczema from July 2019–January 2020. Parents were interviewed face to face at the University of Southampton or in participants’ homes, or via telephone, depending on their preferences. All interviews were audio-recorded. Participants received a £20 voucher for their time.

Box 1 **Summary of interview guide, comprising eight broad questions and accompanying prompts**

1. I’ve got quite a broad question to start with. Please can you tell me a bit about your experiences of going online about your child’s eczema?
  - When did you first look at online resources?
  - When do you typically look at online resources now?
  - What made you choose to look at online resources?
2. Thinking back to the last time you used [insert online resources they might have already said that they use] relating to your child’s eczema, can you remember what you were looking for?
  - How typical is [insert what they said they looked for] in terms of what you tend to look for online?
  - What other things have you looked for on this/these websites or similar?
3. [If not mentioned already] can you tell me about what types of treatments for eczema you have searched about online?
  - Which online resources?
  - Any other types of treatments?
  - Ask about steroids
4. Please tell me about how you have used the advice and support you get online?
  - Do you think you’ve changed how you’ve thought about or managed your child’s eczema, based on something you’ve read about eczema treatments online?
5. How do you decide what to look at online?
  - How do you decide what’s helpful or not helpful to you?
  - Do you find there’s a lot of stuff to look at online or not much?
  - Discussing the online information with health professionals or friends and/or family?
6. Can you tell me about what sort of things you’ve discussed about with other parents and carers online (that is, through social media sites, blogs, and/or online discussion forums)?
  - Have you ever shared your own experiences or advice?
  - [if not mentioned already] have you ever shared your experiences or given advice about eczema treatments?
  - What were other people’s responses like? Or ‘why not’?
7. Please tell me anything else about your views and experiences of using online resources for advice and support about eczema treatments, and anything else that might further help health professionals and researchers to learn about the role of the internet for parents and carers of children with eczema
8. Is there anything else you’d like to say before we finish up?

### Data processing and analysis

Interviews were audio-recorded and transcribed verbatim. NVivo (version 12) facilitated analysis. To protect the anonymity of participants, all identifying information was removed, and participants’ names were replaced with identification numbers. Data were stored securely in a password-protected file on a University of Southampton server, which only authorised staff members were able to access.

Interview transcripts were analysed inductively using reflexive thematic analysis, consisting of six flexible and recursive phases.^18,19^ In phase 1, BT listened to the audio-recordings and noted initial interpretations for codes in transcripts. In phase 2, BT inductively coded transcripts line-by-line, according to their semantic meaning, until coding reached saturation. In phases 3, 4, and 5, BT developed and refined a list of sub-themes, together with IM, ET, NC, and MS. They combined similar codes and retained individual codes as sub-themes, and grouped together sub-themes surrounding similar topics to form overarching themes. While coding transcripts, BT reflected on her limited experience of managing eczema, therefore themes and sub-themes were further refined together with AR, a public contributor with experience caring for children with eczema and using online parenting forums. With the aims of the study in mind, BT re-examined all coded data to assess fit within the developed sub-themes and overarching themes, and amended where necessary. BT developed a coding manual for transparency purposes, and in phase six wrote up the findings together with the whole research team.

### Patient and public involvement

Input from two parents of children with eczema experienced in using online parenting forums for eczema advice informed the focus of the interview guide and wording in the patient information sheet. Suggestions included using accessible language, acknowledging that parents of children with eczema are experts in their own experiences, and considering the importance of social support from online resources.

## Results

### Participant characteristics

Fifteen parents of children with eczema participated in semi-structured qualitative interviews. All participants were mothers (*n* = 15), aged between 26 and 50 years, and the majority of whom lived in England (*n* = 14). Most participants had one child at home with eczema (*n* = 11), aged between 1 year and 15 years. Most participants were recruited opportunistically or through snowballing (*n* = 8), and interviewed via telephone (*n* = 10). See Table 1 for participant characteristics.

**Table 1.** Characteristics of participants who took part in qualitative interviews about making sense of and acting on advice about eczema treatments in online forums

Gender	*n*	Children with eczema in household	*n*
Female	15	1	11
Male	0	2	4
		≥3	0
**Age**	***n***		
18–25 years	0	**Age of child(ren) with eczema**	***n***
26–35 years	6	0–2 years	6
36–50 years	9	3–5 years	7
>51 years	0	6–8 years	3
		9–11 years	1
**Residency**	***n***	12–15 years	2
England	14	16–18 years	0
Scotland	1		
Wales	0	**Recruitment method**	***n***
Northern Ireland	0	Online advertisement	7
		Opportunistic or snowballing	8
**Format of interview**	***n***		
Face to face at university	4		
Face to face at home	1		
Telephone	10		

### Overarching themes

**Figure 1.**
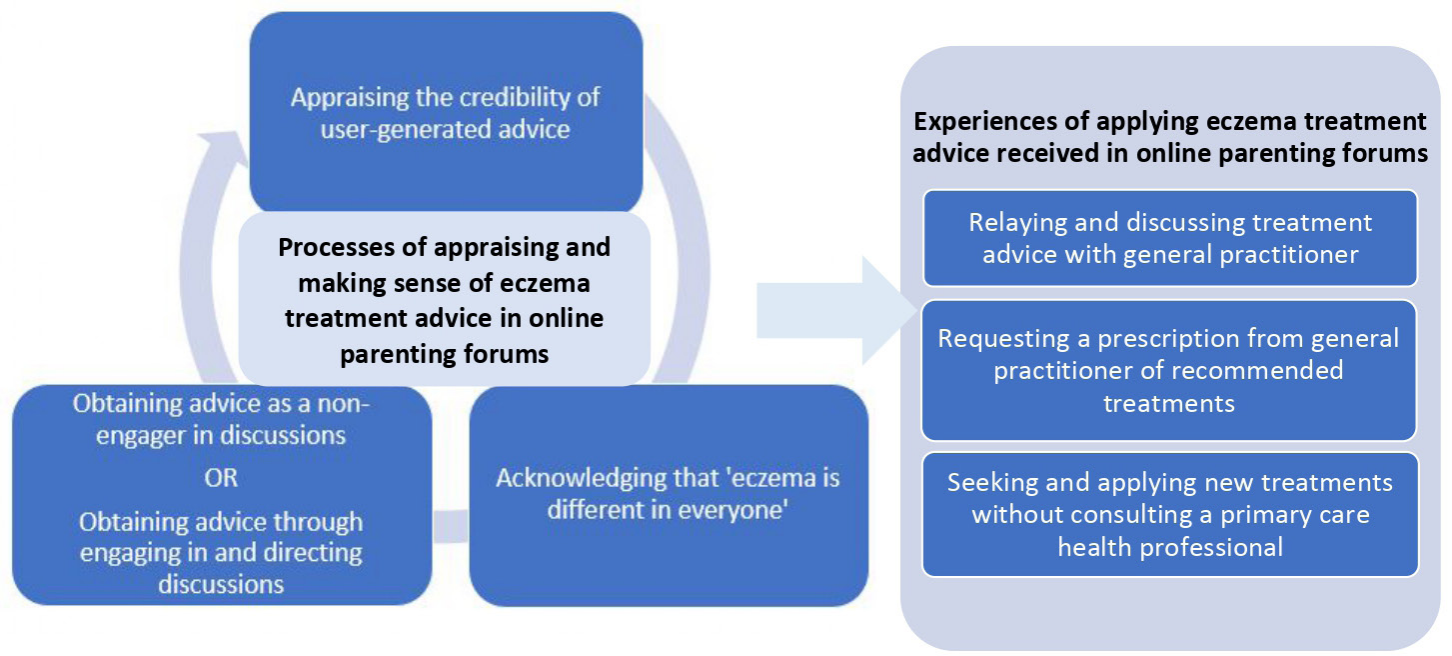
Overview of themes and sub-themes about parents' experiences of making sense of and acting on eczema treatment advice found in online parenting forums

The following two overarching themes were developed from the data: (1) Processes of appraising and making sense of eczema treatment advice in online parenting forums; and (2) Experiences of applying eczema treatment advice received in online parenting forums. There were six sub-themes, which explained how parents of children with eczema made sense of and acted on advice about eczema treatments found in online parenting forums (Figure 1).

### Processes of appraising and making sense of eczema treatment advice in online parenting forums

Many parents reflected on appraising the credibility of user-generated advice in online parenting forums, before attempting to make sense of it, and potentially using it later. Parents reported appraising the trustworthiness of advice through comparing advice from one online resource with another online resource to check for consistencies, and considering the terminological accuracy of the language used:

*'There is language that medically trained people use and then there is language that other people don't, so I guess when I read something online you have a bit of a like of a light flashes when there is terminology that just sounds not factually correct, and there is other stuff that just sounds like it’s scaremongering.'* (Participant 5, mother of child with eczema aged 3 years)

After appraising the credibility of other parents’ advice about eczema treatments in online parenting forums and beginning to make sense of the advice, several parents reported progressing onto acknowledging that ‘eczema is different in everyone’. Parents explained that because eczema manifests and responds to treatment differently in different people, parents in online parenting forums inevitably express varying beliefs surrounding eczema treatments. Parents noted that it is important for them to remember that treatments recommended or discouraged in online parenting forums may not necessarily work or not work for their child(ren), and to thus take advice at arm’s length:

'[a popular pharmaceutical emollient] *was another massive one that a lot of people recommended because it’s to do with oats and stuff like that, which are really good with children for eczema, but it just did not work with* [child’s name]. *Obviously, I'm not slating these companies because they do work for other people, but with my daughter they just don't.'* (Participant 11, mother of child with eczema aged 18 months)

It appeared that once parents had decided to make sense of and comprehend the treatment advice received in online parenting forums following appraising its credibility and suitability, they either: remained as an outsider to the discussion, obtaining advice as a non-engager in discussions; or delved in, obtaining advice through engaging in and directing discussions. Parents who retained advice through simply reading it and not engaging in discussions reported making sense of advice by themselves in this way, without further exploring and discussing concepts with other parents online:

*'I'll type in what I'm looking for in the group, and if someone’s already written about it then I'd read that, so I wouldn't post, I'd read first, and then I'd go and do substantial research on what they've put down to back up what I'm thinking as a mother.'* (Participant 9, mother of child with eczema aged 18 months)

Some parents described how they had read advice in the forums for interest, without intent of acting on it. Parents who made sense of advice through actively engaging and contributing to discussions in online parenting forums described how they often started discussions or engaged in existing conversations in order to ask relevant questions to help them to make sense of other parents’ advice:

*'These people have the same sort of issues and it gives you the opportunity to ask a very specific question and then people who know the answer will answer.'* (Participant 6, mother of child with eczema aged 3 years)

### Experiences of applying eczema treatment advice received in online parenting forums

Many parents reported that after they had taken on board advice about eczema treatments from other parents in online parenting forums, they recalled relaying and discussing treatment advice with GPs. Some parents appeared to feel comfortable taking advice read online into consultations. Parents explained that they brought up the online treatment advice to further discuss and make sense of such advice, which could be helpful for them and their child. They felt that this helped facilitate discussions in consultations and consequently their understanding of eczema management:

*'With your GP lots of things get said and it can be quite hard to process so going online and taking bits of knowledge about treatment methods, I can take those into those situations.'* (Participant 6, mother of child with eczema aged 3 years)

Some parents suggested that they avoided discussing advice obtained in online parenting forums with their GP, because of their perception that their practitioner did not support use of online resources:

*'I don't think they like hearing it, do they? I don't think GPs like hearing that you've been Googling things and doing things, I think they just wash over it, don't they? I don't think they're keen on hearing that kind of thing!'* (Participant 10, mother of child with eczema aged 3 years)

Other parents reported not agreeing with their GP’s advice after reading conflicting messages in online parenting forums:

*'My doctor, when I first went to him, announced that it was definitely a food allergy that was causing it, which I now believe not to be true, from what I've read. I haven't been back to that particular doctor, but I think I might, having been a bit more informed back then, have said, "Why do you think it’s a food allergy?" I feel like I might have said to him, "Look, I've seen this online. It’s not necessarily a food allergy, so stop."'* (Participant 1, mother of child with eczema aged 1 year)

Several parents also described requesting a prescription from the GP for a treatment recommended in the online forum. Some parents recalled successful experiences, and reported feeling that their GP had listened to them:

*'Someone on my* [social media website] *page … she had said, "[pharmaceutical emollient]." When I spoke to the doctor about it, they were like, "Okay, yes, we can prescribe that."' (Participant 11, mother of child with eczema aged 18 months*)

Other parents reported that their GPs were not interested or were otherwise opposed treatment requests that parents had brought into consultations based on advice in online parenting forums:

*'There was a recent one that’s come out about a* [online doctor] *treatment, or something. I was like, what the hell is this? Then, I talked to my doctor and he was like, "Absolutely, under no circumstances."*' (Participant 2, mother of children with eczema aged 6 and 15 years)

Another common way in which parents acted on treatment advice received in online parenting forums was through seeking and applying new treatments without consulting a primary care health professional. Experiences included applying topical treatments differently, writing down treatment instructions, amending their child’s diet, and purchasing over-the-counter treatments and internet-bought treatments. Some parents appeared to recall feeling relatively comfortable starting treatments without consulting a clinician. Some reported successful experiences:

*'I went online and they recommended something this green, organic actual oat bath. I can't believe that. I've completely missed that out. I actually went into* [supermarket]*. They were selling it in there and it’s the best product I've ever used. It’s fantastic.*' (Participant 11, mother of child with eczema aged 18 months)

On the other hand, others reported unsuccessful experiences:

*'You get a lot of people saying, "Oh, you need to take out milk" or, "You need to take out wheat"… I did look to eliminate milk. We tried a hydrolysed formula ... It didn't make any difference.'* (Participant 8, mother of child with eczema aged 8 years)

Conversely, some parents recalled avoiding treatments recommended in online parenting forums, because they deemed the treatments not to be suitable for their child:

*'Also, online, there’s a lot of people selling their miracle potions that, unfortunately for parents like myself with children with extreme eczema — I, personally, haven't bought them …'* (Participant 2, mother of children with eczema aged 6 and 15 years)

## Discussion

### Summary

The aim of this study was to explore how parents of children with eczema made sense of and acted on advice concerning eczema treatments in online parenting forums. This study has suggested that when seeking treatment advice from online parenting forums, parents may start by appraising the trustworthiness and relevance of advice before progressing on to reading, engaging with, and later acting on it. Some parents expressed that they made sense of online advice through only reading content, whereas others read and engaged in online discussions in order to direct topics or seek advice most relevant to them and their child. During the sense-making process, parents appeared mindful of the fact that treatments that have worked for others may not work for their child, thus thought rationally before potentially acting on the advice. Parents of children with eczema often reported acting on advice that they read in online forums. In particular, parents described how they had brought up advice received online in subsequent consultations with their GP to further make sense of it and to request prescriptions of treatments recommended in online forums. On other occasions, parents described trying new treatments without consulting a clinician, or otherwise avoiding treatments recommended online when the advice appeared untrustworthy or unsuitable for their child.

### Strengths and limitations

A varied sample of mothers of children with eczema was obtained in terms of the age of their child or children with eczema, and the severity of their child or children’s eczema. The broad range of experiences that were shared around making sense of and acting on eczema advice in online parenting forums solidified the rationale for exploring this phenomenon. Saturation was reached during the coding process of the thematic analysis, which strengthened the reliability of the developed overarching themes and sub-themes.

A limitation of this study was the relatively small sample size, and the fact that all participants were mothers. Potentially different insights from a larger sample size, including insights from fathers with eczema advice in online parenting forums were not obtained. Another limitation was that demographic data were not collected about participants’ ethnic group nor relative deprivation. This meant that potential differing patterns could not be explored between parents of various ethnic groups and deprivation levels in terms of how they made sense of and acted on advice. Additionally, parents’ experiences were mostly retrospective accounts and a different study design, such as longitudinal data collection, might have provided different insights into the ways parents act on information found online. A final limitation is that the study did not purposively explore parents’ experiences of discussing advice with different types of primary care health professionals, which could have revealed potential differences in parents’ experiences associated with specific health professional roles. Parents in this study mostly discussed experiences with GPs, and the study was conducted before the COVID-19 pandemic. Therefore, since this study was conducted, and owing to the increased responsibilities and roles of allied primary care health professionals since the COVID-19 pandemic, parents today may have more experiences of discussing advice with and relaying advice from a variety of primary care health professionals.

### Comparison with existing literature

A previous interview study with parents of children with eczema found that parents felt confident about assessing whether advice obtained online was useful to them or not.^10^ This was not explicitly articulated in the current study, although many parents reported specific processes for appraising advice in online parenting forums, suggesting that they were at least somewhat confident in their ability to do so. In comparison, parents of children with other long-term and acute conditions have expressed not feeling confident in their ability to appraise the trustworthiness of online advice.^13,14^ Similar processes for determining the quality of online content have been found in other people seeking online advice around health and illness, including looking for consensus across multiple information resources, and appraising the language used.^15^ Previous reviews of parents of children with other long-term and acute conditions found that personal situations, such as symptoms and needs, prior knowledge or experience of a source, personal knowledge and beliefs, and intuition, influenced how they appraised online advice. Personal situations were not articulated in the current study, but may provide some insight into the processes through which parents of children with eczema appraise the credibility of eczema treatment advice.

Previous studies exploring the discussions of people living with breast cancer, fibromyalgia, and arthritis,^20^ HIV and AIDS,^21^ and infertility^22^ in online forums similarly distinguished between people who only read online advice and people who actively post and engage in online discussions. A previous study of discussions between parents of children with eczema in online parenting forums,^11^ and the wider literature of parents of children with other long-term and acute conditions^14^ and adults living with various illnesses,^16^ have also uncovered that people follow-on with advice received from online forums. They follow-on by scheduling appointments with health professionals, facilitating discussions in subsequent medical consultations, and requesting certain treatments. The wider literature has similarly found that people test self-management strategies discussed by others in online forums.^16,17^ A contrast between the findings from the current study and previous literature is that parents of children with eczema in the current study did not report forming friendships offline after meeting in online parenting forums, unlike other people who have sought health and illness advice from online forums.^16,17^ This difference is potentially because parents have less time to develop in-person friendships when caring for children.

This is the first study to suggest how parents of children with eczema make sense of and act on eczema treatment advice exchanged in online parenting forums. This study builds on existing research exploring parents of children with eczema and online parenting forums,^11–13^ through highlighting that parents undergo a progressive process of starting with an appraisal of credibility and relevance of advice, before either just reading advice or actively engaging in discussions, and before potentially acting on and applying the advice to manage their child’s eczema. These experiences have not previously been explored in this population.

### Implications for research and practice

Future research could explore whether the impact of the COVID-19 pandemic on receiving timely appointments with primary care health professionals has led to more parents of children with eczema turning to advice from online parenting forums, as opposed to waiting for an appointment. Informing health professionals of these findings — that parents of children with eczema often appraise the trustworthiness of online advice, that some make sense of advice by simply reading it, and that others prefer engaging in discussions — could encourage clinicians to provide more advice and/or clarity, depending on the sense-making preference of the parent, and to signpost parents to reliable online resources. Advising primary care health professionals that some parents might subsequently discuss online advice in consultations and ask for specific treatments, and that some will try new treatments that are recommended online and not necessarily discuss this with them, could encourage health professionals to ask questions that they might not have originally considered. This could improve professional–parent discussions and ultimately improve parents’ management of their child’s eczema. Awareness of parents’ processes of appraising and making sense of online advice around eczema treatments could also be beneficial for researchers designing interventions for the management of childhood eczema, who can consider including credible advice, a mixture of read-only and engaging content, experiential information from other parents, and advice about subsequently using the information to manage childhood eczema.

## Data availability

None available.

## Data

None available.
